# Supplementary material for: Beyond Analgesia: Psychobiotics as an Adjunctive Approach to Pain Management in Gastrointestinal Oncology—A Post Hoc Analysis from the ProDeCa Study
Source: Nutrients. 2025 Aug 25;17(17):2751. doi: 10.3390/nu17172751 (PMC12430046; doi:10.3390/nu17172751)
Supplement: Supplementary file 1 [file nutrients-17-02751-s001.zip › nutrients-3785294-supplementary.pdf]

Supplementary Table 1: Demographic characteristics

	Psychobiotics		p-value	Placebo		p-value
**Number of patients**	45			54		
**Depression Status**	Non-Depressed (n=27)	Depressed (n=18)		Non-Depressed (n=31)	Depressed (n=23)	0.794
Age (years-median-IQR)	65.4 (11.6)	58.7 (14.9)	0.246	63.6 (9.0)	68.4 (13.8)	0.033
Gender (M/F)	12/15	17/1	0.001	18/13	13/10	0.910
Weight (kg)	76.1 (15.1)	74.1 (12.2)	0.546	69.3 (15.7)	72.4 (9.2)	0.478
Height (cm)	164.6 (7.7)	173.8 (6.9)	<0.001	170.9 (9.1)	165.7 (7.7)	0.039
BMI (Kg/m^2)	28.1 (5.3)	24.6 (4.1)	0.041	23.6 (4.5)	23.4 (2.7)	0.020
Weight Loss (Yes/No)	21/6	16/2	0.340	24/7	14/9	0.188
Marity Status						
Free	8	2		6	2	
Married	11	9	0.224	22	17	0.146
Divorced/ Widow/er	8	7		3	4	
Education						
Basic	5	3	0.987	9	5	0.570
Secondary	16	11		13	13	
University	6	4		9	5	
Income						
<10K Euros	5	7		13	9	
10-30K Euros	20	11	0.194	15	13	0.705
>30K Euros	2	0		3	1	
Health Insurance						
Public/None	25	14		26	21	
Private	1	0	0.118	3	1	0.706
Both	1	4		2	1	
Habits						
Smoking (Yes/No)	19/8	9/9	0.236	14/17	9/14	0.658
Alcohol (Yes/No)	4/23	6/12	0.143	3/28	3/20	0.697
Sedatives (Yes/No)	9/18	5/13	0.693	8/23	6/17	0.981
Tumor Location						
Gastric	6	4		7	6	
Large Bowel	13	9	0.998	14	12	0.718
Rectum	5	3		8	3	

Pancreas	3	2		2	2	
Disease stage<br>Stage I<br>Stage II<br>Stage III<br>Stage IV	 3<br>11<br>9<br>4	 2<br>9<br>5<br>2	0.934	 3<br>13<br>10<br>5	 2<br>10<br>8<br>3	0.988
Charlson Comorbidity Index (median-IQR)	4.0 (1.0)	4.5 (1.5)	0.896	4.0 (1.0)	4.0 (1.5)	0.913
Side-effects of chemo-:<br>Diarrhea (Yes/No)<br>Vomiting (Yes/No)<br>Chemo- temporary dis-continuation (Yes/No)<br>Hair Loss (Yes/No)	 8/19<br>4/23<br>2/25<br>1/26	 6/12<br>3/15<br>1/17<br>1/17	 0.792<br>0.867<br>0.807<br>0.768	 8/23<br>5/26<br>2/29<br>2/29	 7/16<br>3/20<br>2/21<br>1/22	 0.707<br>0.752<br>0.756<br>0.739

Supplementary Table 2 presents the descriptive statistics of the qualities of pain:

pain rating index of each one of the 15 items in the 3 time-points, per group of participants.

Non-depressed taking psychobiotics

		T0 (n=27)						T1 (n=27)						T2 (n=24)					
Item	Range	min	max	mean	SD	median	IQR	min	max	mean	SD	median	IQR	min	max	mean	SD	median	IQR
1	0-3	0	3	1.48	1.28	1.00	3.00	0	3	0.85	0.99	1.00	2.00	0	3	0.75	0.99	0.00	1.00
2	0-3	0	3	1.44	1.15	2.00	2.00	0	3	1.37	1.08	2.00	2.00	0	3	0.96	0.95	1.00	2.00
3	0-3	0	3	1.19	1.11	1.00	2.00	0	3	1.15	0.95	1.00	2.00	0	2	0.96	0.95	1.00	2.00
4	0-3	0	3	1.41	1.19	2.00	2.00	0	3	0.89	0.93	1.00	2.00	0	2	0.54	0.78	0.00	1.00
5	0-3	0	3	1.22	1.05	1.00	2.00	0	2	0.89	0.89	1.00	2.00	0	2	0.67	0.87	0.00	1.75
6	0-3	0	3	1.00	1.04	1.00	2.00	0	2	0.85	0.95	0.00	2.00	0	2	0.67	0.92	0.00	2.00
7	0-3	0	3	1.26	1.20	1.00	2.00	0	3	1.07	0.96	1.00	2.00	0	2	0.92	0.93	1.00	2.00
8	0-3	0	3	1.19	1.04	1.00	2.00	0	2	1.04	0.98	1.00	2.00	0	2	1.00	0.98	1.00	2.00
9	0-3	0	3	1.41	1.28	2.00	3.00	0	3	1.19	1.04	1.00	2.00	0	2	0.88	0.95	0.50	2.00
10	0-3	0	2	0.44	0.80	0.00	1.00	0	2	0.22	0.58	0.00	0.00	0	1	0.08	0.28	0.00	0.00
11	0-3	0	3	1.19	1.30	1.00	3.00	0	3	0.85	1.06	0.00	2.00	0	2	0.71	0.86	0.00	1.75
12	0-3	0	3	1.26	1.13	1.00	2.00	0	3	1.04	1.02	1.00	2.00	0	2	0.88	0.90	1.00	2.00
13	0-3	0	3	1.30	1.27	1.00	3.00	0	3	1.04	1.06	1.00	2.00	0	3	0.88	0.99	0.50	2.00
14	0-3	0	3	1.44	1.09	2.00	2.00	0	3	1.37	1.04	2.00	2.00	0	3	1.29	1.04	1.00	2.00
15	0-3	0	3	1.44	1.37	1.00	3.00	0	3	1.26	1.35	1.00	3.00	0	3	0.92	1.18	0.00	2.00

Non-depressed taking placebo

		T0 (n=31)						T1 (n=31)						T2 (n=27)					
Item	Range	min	max	mean	SD	median	IQR	min	max	mean	SD	median	IQR	min	max	mean	SD	median	IQR
1	0-3	0	3	1.29	1.07	1.00	2.00	0	3	1.26	1.03	1.00	2.00	0	3	1.37	1.15	1.00	3.00
2	0-3	0	3	1.26	0.96	2.00	2.00	0	3	1.26	1.00	2.00	2.00	0	3	1.52	1.09	2.00	2.00
3	0-3	0	3	1.03	1.02	1.00	2.00	0	3	1.13	1.06	1.00	2.00	0	3	1.52	1.19	2.00	2.00
4	0-3	0	3	0.97	1.05	1.00	2.00	0	3	1.32	1.17	1.00	2.00	0	3	1.37	1.11	1.00	2.00
5	0-3	0	3	1.39	1.02	2.00	2.00	0	3	1.45	1.09	2.00	2.00	0	3	1.74	1.13	2.00	2.00
6	0-3	0	3	1.35	0.98	2.00	2.00	0	3	1.42	0.99	2.00	1.00	0	3	1.52	1.12	2.00	2.00
7	0-3	0	3	1.19	1.01	1.00	2.00	0	3	1.42	0.85	2.00	1.00	0	3	1.59	1.01	2.00	1.00
8	0-3	0	3	1.03	1.08	1.00	2.00	0	3	1.10	1.14	1.00	2.00	0	3	1.22	1.25	2.00	2.00
9	0-3	0	3	1.03	1.11	0.00	2.00	0	3	1.03	1.11	0.00	2.00	0	3	1.11	1.05	2.00	2.00
10	0-3	0	2	0.32	0.65	0.00	0.00	0	3	0.32	0.75	0.00	0.00	0	2	0.52	0.85	0.00	1.00
11	0-3	0	3	1.03	1.11	1.00	2.00	0	3	0.87	1.06	0.00	2.00	0	3	1.11	1.22	1.00	2.00
12	0-3	0	3	0.97	1.05	1.00	2.00	0	3	0.97	1.05	1.00	2.00	0	3	1.22	1.22	1.00	2.00
13	0-3	0	3	1.03	1.08	1.00	2.00	0	3	0.97	1.08	1.00	2.00	0	3	1.15	1.17	1.00	2.00
14	0-3	0	3	1.06	1.21	0.00	2.00	0	3	0.94	1.21	0.00	2.00	0	3	1.18	1.30	0.00	2.00
15	0-3	0	3	1.03	1.25	0.00	2.00	0	3	1.00	1.21	0.00	2.00	0	3	1.19	1.27	1.00	2.00

Depressed taking psychobiotics

		T0 (n=18)						T1 (n=18)						T2 (n=15)					
Item	Range	min	max	mean	SD	median	IQR	min	max	mean	SD	median	IQR	min	max	mean	SD	median	IQR
1	0-3	0	2	0.56	0.62	0.50	1.00	0	3	0.50	0.79	0.00	1.00	0	2	0.47	0.64	0.00	1.00
2	0-3	0	3	1.11	1.08	1.50	2.00	0	3	0.89	1.02	0.50	2.00	0	3	0.67	0.98	0.00	1.00
3	0-3	0	3	1.06	1.00	1.00	2.00	0	2	0.61	0.78	0.00	1.00	0	3	0.47	0.92	0.00	1.00
4	0-3	0	3	1.00	1.19	0.00	2.00	0	3	0.72	1.07	0.00	2.00	0	2	0.67	0.98	0.00	2.00
5	0-3	0	3	0.89	0.96	1.00	2.00	0	3	0.94	0.94	1.00	2.00	0	2	0.73	0.80	1.00	1.00
6	0-3	0	3	0.94	1.00	1.00	2.00	0	3	0.94	1.00	1.00	2.00	0	3	0.87	1.06	0.00	2.00
7	0-3	0	2	0.50	0.79	0.00	1.00	0	3	0.39	0.78	0.00	1.00	0	1	0.20	0.41	0.00	0.00
8	0-3	0	2	0.33	0.77	0.00	0.00	0	1	0.17	0.38	0.00	0.00	0	1	0.20	0.41	0.00	0.00
9	0-3	0	3	0.72	1.02	0.00	2.00	0	2	0.61	0.92	0.00	2.00	0	2	0.67	0.90	0.00	2.00
10	0-3	0	1	0.17	0.38	0.00	0.00	0	2	0.33	0.77	0.00	0.00	0	2	0.13	0.52	0.00	0.00
11	0-3	0	3	0.39	0.92	0.00	0.00	0	2	0.22	0.65	0.00	0.00	0	2	0.27	0.70	0.00	0.00
12	0-3	0	2	0.33	0.77	0.00	0.00	0	2	0.28	0.67	0.00	0.00	0	2	0.27	0.59	0.00	0.00
13	0-3	0	3	0.50	0.92	0.00	1.00	0	2	0.50	0.71	0.00	1.00	0	1	0.33	0.49	0.00	1.00
14	0-3	0	3	0.72	1.02	0.00	2.00	0	3	0.56	0.98	0.00	1.25	0	3	0.67	0.98	0.00	1.00
15	0-3	0	3	0.56	0.86	0.00	1.00	0	2	0.44	0.70	0.00	1.00	0	2	0.47	0.64	0.00	1.00

Depressed taking placebo

		T0 (n=23)						T1 (n=23)						T2 (n=17)					
Item	Range	min	max	mean	SD	median	IQR	min	max	mean	SD	median	IQR	min	max	mean	SD	median	IQR
1	0-3	0	3	1.13	1.10	1.00	2.00	0	3	1.13	1.10	1.00	2.00	0	3	1.71	1.16	2.00	2.00
2	0-3	0	2	1.04	0.98	1.00	2.00	0	3	1.17	1.03	1.00	2.00	0	3	1.71	1.16	2.00	2.50
3	0-3	0	3	0.74	1.00	0.00	2.00	0	3	0.83	1.07	0.00	2.00	0	3	1.41	1.06	2.00	2.00
4	0-3	0	3	0.65	1.11	0.00	1.00	0	2	0.43	0.79	0.00	1.00	0	3	0.71	1.16	0.00	2.00
5	0-3	0	3	1.13	1.01	1.00	2.00	0	2	1.04	0.88	1.00	2.00	0	3	1.12	0.99	1.00	2.00
6	0-3	0	3	0.65	0.83	0.00	1.00	0	2	0.57	0.66	0.00	1.00	0	2	0.82	0.88	1.00	2.00
7	0-3	0	1	0.30	0.47	0.00	1.00	0	2	0.52	0.79	0.00	1.00	0	2	0.59	0.80	0.00	1.00
8	0-3	0	3	0.61	1.03	0.00	1.00	0	3	0.70	1.02	0.00	1.00	0	3	0.59	0.94	0.00	1.00
9	0-3	0	3	0.91	1.04	0.00	2.00	0	3	1.04	1.07	1.00	2.00	0	2	1.06	1.03	2.00	2.00
10	0-3	0	2	0.26	0.54	0.00	0.00	0	2	0.26	0.54	0.00	0.00	0	1	0.24	0.44	0.00	0.50
11	0-3	0	2	0.39	0.78	0.00	0.00	0	3	0.52	1.04	0.00	0.00	0	3	0.59	1.12	0.00	1.00
12	0-3	0	2	0.61	0.94	0.00	2.00	0	3	0.65	1.03	0.00	1.00	0	3	0.94	1.20	0.00	2.00
13	0-3	0	2	0.61	0.72	0.00	1.00	0	2	0.52	0.67	0.00	1.00	0	2	0.94	0.83	1.00	2.00
14	0-3	0	3	0.65	0.93	0.00	1.00	0	3	1.00	1.13	0.00	2.00	0	3	1.00	1.12	0.00	2.00
15	0-3	0	3	0.57	1.04	0.00	1.00	0	3	0.52	0.95	0.00	1.00	0	3	0.71	1.05	0.00	1.00

Horizontal line between items 11 and 12 is to separate sensory pain descriptors [items 1-11] and affective pain descriptors [items 12-15] -see below
